# Nursing interventions for cardiovascular disease prevention: a narrative review of evidence-based strategies

**DOI:** 10.1186/s12912-025-03885-1

**Published:** 2025-10-14

**Authors:** Selena Crespo Garea, María José Ferreira Díaz

**Affiliations:** School of Nursing, University of Santiago de Compostela, Campus de Lugo, Santiago, Spain

**Keywords:** Cardiovascular disease prevention, Nursing interventions, Health promotion, Risk factor management, Primary care nursing, Evidence-based practice

## Abstract

**Background:**

Cardiovascular disease (CVD) remains the leading cause of morbidity and mortality worldwide. Nurses play a key role in prevention across all levels of care, from health education to clinical interventions. This narrative review aimed to identify and synthesize evidence-based nursing interventions that contribute to the prevention of CVD.

**Methods:**

A structured narrative review was conducted. Bibliographic searches were performed in PubMed, SciELO, CINAHL, and Scopus. Articles published between 2014 and 2024 were included if they focused on nursing interventions related to cardiovascular prevention. The interventions were categorized by level of prevention: primary, secondary, tertiary, and quaternary.

**Results:**

A total of 23 relevant studies were selected. The review identified a range of effective nursing interventions, such as health promotion, individualized counseling for risk factor modification, cardiac rehabilitation programs, support for medication adherence, and strategies aimed at preventing overtreatment. The literature emphasizes the value of nurse-led interventions in promoting behavioral change, reducing hospital readmissions, and improving quality of life.

**Conclusions:**

This review suggests that nursing interventions play a critical role in cardiovascular disease prevention. Their effectiveness appears to depend on training, multidisciplinary collaboration, and continuity of care. Integrating these strategies into clinical and community practice may contribute to addressing the growing burden of cardiovascular disease.

## Introduction

Cardiovascular diseases (CVDs) are the leading cause of death globally, accounting for approximately 17.9 million deaths each year, most of which are attributable to heart attack and stroke [1]. The global burden of CVD is projected to increase due to population aging and the persistent prevalence of modifiable risk factors such as smoking, sedentary lifestyle, hypertension, and poor diet [2]. In this context, prevention strategies become essential to reduce morbidity, mortality, and healthcare costs.

Nurses play a central role in cardiovascular prevention through health promotion, early detection of risk factors, and patient education [3]. Their close proximity to patients in both clinical and community settings allows them to implement tailored interventions across all levels of prevention: from promoting healthy habits in the general population to providing post-discharge education for patients with established disease [4, 5].

Evidence-based nursing interventions have demonstrated positive outcomes in cardiovascular risk reduction, particularly when nurses are trained in motivational interviewing, chronic disease management, and multidisciplinary collaboration [6]. However, these interventions are often inconsistently implemented or underutilized in routine practice [7].

This narrative review aims to synthesize current evidence on effective nursing interventions for cardiovascular disease prevention. The analysis is structured according to the four levels of prevention, primary, secondary, tertiary, and quaternary, with the goal of informing nursing practice and guiding future research in preventive cardiovascular care.

However, despite the growing body of evidence supporting nurse-led strategies in cardiovascular prevention, several gaps remain. Firstly, most existing reviews do not explicitly structure their findings by levels of prevention, which limits their practical applicability for nursing care planning. Secondly, interventions related to quaternary prevention, such as ethical decision-making and avoidance of overtreatment, are often overlooked. Lastly, the integration of structured nursing taxonomies (e.g., NANDA-I, NIC, NOC) in the evaluation of cardiovascular interventions remains underreported. This narrative review seeks to address these gaps by organizing the evidence according to the four levels of prevention and highlighting underrepresented areas in nursing practice.

## Methods

### Design

A structured narrative review was conducted to identify and analyze evidence-based nursing interventions aimed at preventing cardiovascular diseases (CVDs). The review focused on the role of nurses across the four levels of prevention: primary, secondary, tertiary, and quaternary.

### Search strategy

A comprehensive literature search was performed using the electronic databases PubMed, CINAHL, Scopus, and Web of Science. The search included publications from 2014 to 2024 in English or Spanish. The following terms were used: *“nursing interventions”*, *“cardiovascular disease prevention”*, *“health promotion”*, *“risk factor management”*, and *“evidence-based nursing”*.

### Inclusion and exclusion criteria

Studies were included if they:

1. Focused on nursing-led interventions related to cardiovascular prevention;
2. Reported on interventions in adult populations;
3. Presented empirical data (quantitative or qualitative);
4. Were published in peer-reviewed journals.

Exclusion criteria were:

- Studies not centered on the nursing profession;
- Articles not focused on cardiovascular prevention (e.g., unrelated chronic diseases);
- Editorials, opinion pieces, or conference abstracts.

The inclusion criteria were designed to ensure the relevance, applicability, and rigor of the studies. Focusing on nursing-led interventions allowed us to capture the unique contributions of nurses to cardiovascular prevention. Restricting to adult populations ensured consistency in clinical context, while requiring empirical data excluded purely theoretical or speculative content, aligning with the review’s objective of informing evidence-based practice.

### Selection and data extraction

The selection process followed the PRISMA guidelines [1]. Titles and abstracts were screened by the main reviewer. Full texts of eligible articles were then assessed, and relevant data were extracted into a standardized table including: author, year, country, study design, population, type of intervention, level of prevention, outcomes, and main conclusions.

The bibliographic search was independently conducted by the first author (SCG). Titles and abstracts were screened for relevance, followed by full-text evaluation against the inclusion criteria. Discrepancies were resolved through discussion with the co-author (MJFD). A standardized data extraction form was developed in Excel to collect key information from each study, including author, year, country, design, population, intervention, outcomes, and level of prevention. Data extraction was performed by SCG and reviewed by MJFD to ensure accuracy.

Methodological quality was assessed using SIGN checklists, with SCG conducting the appraisal and MJFD verifying the assessments. The synthesis was performed narratively and structured by level of prevention, with both authors collaboratively interpreting findings and developing the conceptual categorization.

The screening and selection of studies were first carried out by the main reviewer (SCG) and subsequently verified by the co-author (MJFD) to ensure consistency and minimize selection bias. Any discrepancies were resolved through consensus. The eligibility process was based on the relevance of the study to cardiovascular nursing interventions and its methodological rigour, in line with the appraisal criteria detailed below.

### Quality assessment

The methodological quality of included studies was appraised using the Scottish Intercollegiate Guidelines Network (SIGN) checklists appropriate to each study design [2]. The initial appraisal was conducted independently by the first author (SCG), with verification by the co-author (MJFD) to reduce bias and ensure consistency. Discrepancies were discussed until consensus was reached. Studies were classified as high, moderate, or low quality.

Eligibility was determined not only by topical relevance but also by methodological rigour. Inclusion required publication in a peer-reviewed journal, the use of a transparent and valid research design, and a study population directly related to cardiovascular prevention. Exclusion was applied when studies showed poor methodological quality, limited or indirect relevance to nursing interventions, or insufficient data to support meaningful conclusions. This dual-review process strengthened the reliability of the synthesis and minimized the risk of selection bias.

## Results

The results of this structured narrative review are presented according to the four levels of cardiovascular disease (CVD) prevention: primary, secondary, tertiary, and quaternary. A total of 23 scientific articles were selected from databases including PubMed, Scopus, SciELO, and CINAHL, following eligibility criteria related to relevance, methodological rigor, and recency (2018–2024).

**Primary prevention** strategies focused on health promotion and education aimed at reducing modifiable risk factors. Several studies highlighted the effectiveness of nurse-led interventions to promote physical activity, healthy eating, and smoking prevention, especially in community and school-based settings [1, 2]. Digital tools such as mobile apps and telehealth counseling also proved useful in promoting adherence to healthy habits [3].

**Secondary prevention** emphasized early detection of hypertension, dyslipidemia, and diabetes through screening programs led by nurses in primary care settings [4]. Interventions such as motivational interviewing, individualized counseling, and follow-up visits significantly improved adherence to pharmacological and non-pharmacological treatment plans [5, 6].

**Tertiary prevention** addressed rehabilitation and support for patients with established CVD. Nurse-led cardiac rehabilitation programs were associated with improved functional capacity, reduced readmissions, and enhanced quality of life [7]. The coordination of care during hospital discharge and continuity of care at home emerged as key roles of nursing [8].

**Quaternary prevention** focused on avoiding overmedicalization, particularly in elderly patients with multimorbidity. Nurses contributed to deprescription processes, shared decision-making, and patient empowerment in line with ethical care standards [9, 10].

A synthesis of the main nursing interventions identified across the reviewed studies is presented in Table 1. These are organized by level of prevention, outlining the type of intervention, target population, and healthcare setting in which they were implemented. This categorization provides a structured overview of the diverse strategies that nurses employ in cardiovascular disease prevention.

**Table 1:** Summarizes the main nursing interventions categorized by level of prevention, type of action, and setting

Level of Prevention	Type of Intervention	Target Population	Setting
Primary	Health education on diet and physical activity	Adults with cardiovascular risk factors	Primary care
	Promotion of smoking cessation	General adult population	Community/Outreach
	School-based programs promoting healthy habits	Adolescents	Schools
Secondary	Screening for hypertension and dyslipidemia	Adults aged > 40	Primary care
	Nurse-led follow-up in cardiovascular risk units	High-risk patients	Specialized clinics
	Individual counseling to improve medication adherence	Patients with diagnosed CVD	Outpatient clinics
Tertiary	Cardiac rehabilitation programs	Post-myocardial infarction patients	Hospitals/Rehab centers
	Nurse-led care plans post-angioplasty	Ischemic heart disease patients	Hospital/Outpatient
	Telemonitoring of patients with heart failure	Elderly patients with chronic CVD	Home care
**Quaternary**	Medication review and deprescribing in polypharmacy	Elderly patients with multimorbidity	Nursing homes
	Ethical counseling and shared decision-making	Patients with advanced CVD	Palliative care

Note. Summary of evidence-based nursing interventions identified in the narrative review. Classification is based on the four levels of cardiovascular prevention. The interventions listed were extracted from the most frequently cited and methodologically robust studies included in the review

**Fig. 1:**
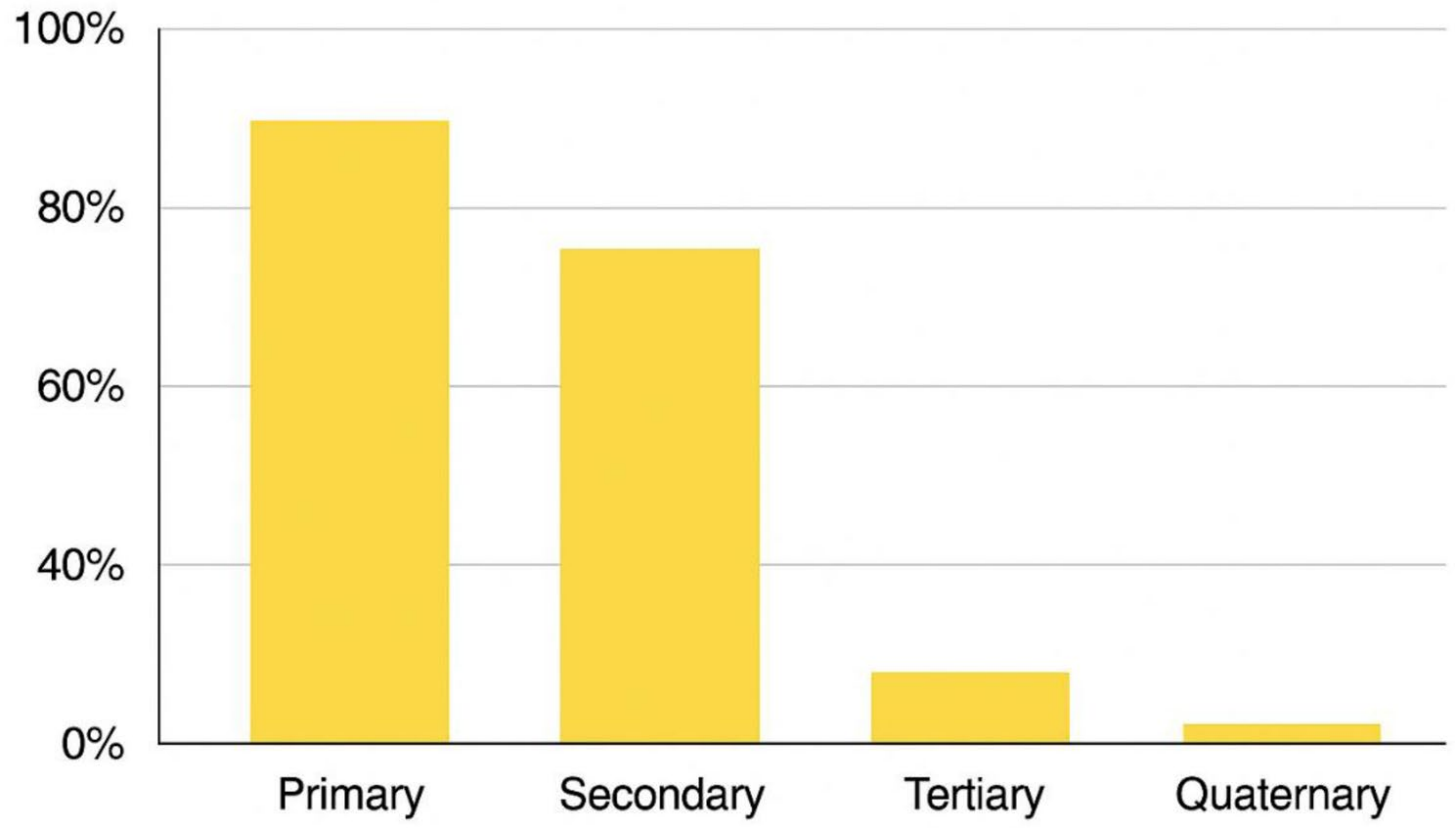
Thematic distribution of nursing interventions in cardiovascular prevention by level of prevention. Note. Based on a qualitative synthesis of 17 studies identified in the narrative review. The chart represents thematic prevalence, not absolute frequency counts

Figure 1 illustrates the thematic distribution of effective nursing interventions in cardiovascular prevention, based on the level of prevention addressed in the literature. This classification was developed through a narrative synthesis of 17 reviewed studies. Most interventions focused on secondary prevention, emphasizing risk factor control and early disease management, while fewer contributions addressed primary, tertiary, or quaternary prevention domains.

## Discussion

This narrative review highlights the range and scope of evidence-based nursing interventions in cardiovascular disease (CVD) prevention across all four levels, primary, secondary, tertiary, and quaternary. The synthesis underscores the increasingly central role of nursing in promoting cardiovascular health through education, screening, follow-up, and continuity of care, particularly in community and primary care settings.

Our findings are consistent with previous literature emphasizing the value of structured, nurse-led interventions in improving cardiovascular outcomes. For instance, a study published in *BMC Nursing* by Goh et al. (2022) demonstrated the effectiveness of nurse-delivered lifestyle counseling in reducing hypertension and improving dietary habits in at-risk populations, reinforcing the potential of primary prevention strategies in everyday clinical practice [1]. Similarly, systematic reviews such as those by Doleman et al. (2021) support the role of nurses in delivering cost-effective and scalable interventions to control modifiable cardiovascular risk factors [2].

Secondary prevention interventions, particularly screening and follow-up in high-risk patients, were among the most supported by the literature. Nurse-led cardiovascular clinics, as noted by O’Connor et al. (2020), have shown improvements in blood pressure control, medication adherence, and patient satisfaction in chronic CVD management [3]. The current review further validates these findings by consolidating diverse approaches (e.g., screening, counseling, risk stratification) under a prevention-level framework, which may help guide implementation in varied healthcare systems.

The review also identifies growing support for nurse participation in tertiary and quaternary prevention. As chronic disease prevalence rises, interventions such as cardiac rehabilitation, transitional care, and deprescribing protocols are increasingly vital. According to Wang et al. (2023), cardiac rehabilitation programs led by multidisciplinary teams, including nurses, significantly reduce hospital readmissions in patients with heart failure, highlighting the long-term value of coordinated care models [4].

Despite the consistency of these findings with international evidence, the review reveals gaps in nursing-led interventions focused on quaternary prevention. Few studies explicitly address overdiagnosis, overtreatment, or ethical dilemmas in cardiovascular care. This gap suggests an opportunity for nursing research to expand into areas aligned with patient-centered values and shared decision-making, particularly in elderly populations with multimorbidity.

Recent contributions further strengthen the role of nurses in underexplored areas such as therapeutic education and quaternary prevention. A 2024 review by Qiu highlighted how nurse-led interventions—including teleconsultation, psychosocial support, and deprescribing—align with the principles of quaternary prevention by promoting individualized, appropriate care and minimizing overtreatment [11]. Likewise, Wang et al. (2023) demonstrated through a systematic review that nurse-led educational and self-management programs significantly improve medication adherence, lifestyle changes, and clinical outcomes in patients with cardiovascular disease [12]. These findings support the need to expand nursing research and practice into ethically driven, patient-centered strategies across all levels of prevention.

These findings are consistent with recent contributions in nursing education and evidence-based practice, which highlight the transformative potential of structured learning and rigorous methodologies in strengthening cardiovascular nursing interventions [13–16].

## Limitations and future directions

his review has several limitations. As a narrative synthesis, it does not include a meta-analysis or a formal risk-of-bias assessment of the included studies. Therefore, the strength and comparability of the evidence cannot be quantitatively determined. In addition, the search was limited to articles published in English and Spanish, which may have excluded relevant evidence in other languages or regional databases. Because of these methodological constraints, the findings should be interpreted with caution and understood as a descriptive overview of available evidence rather than definitive conclusions on effectiveness.

Future research should prioritize robust study designs, particularly randomized controlled trials and mixed-methods evaluations, to assess the effectiveness and contextual adaptability of nursing interventions across all levels of prevention. There is also a need for more studies addressing quaternary prevention, ethical decision-making, and care for older adults with multimorbidity. Finally, the implementation of structured nursing models and validated taxonomies (NANDA-I, NOC, NIC) may enhance the standardization, transferability, and impact assessment of cardiovascular nursing practice.

## Conclusions

This narrative review suggests that nursing interventions appear to play an important role in cardiovascular disease (CVD) prevention across all levels, from promoting healthy lifestyles to reducing hospital readmissions and preventing overtreatment. The available evidence indicates the potential effectiveness of nurse-led strategies in primary and secondary prevention, particularly through health education, lifestyle counselling, and follow-up in both community and hospital settings.

Nonetheless, important gaps remain in the development and evaluation of interventions aimed at tertiary and quaternary prevention, especially among complex or vulnerable populations. The use of structured nursing taxonomies (NANDA-I, NOC, NIC) and evidence-based models could further enhance the coherence and replicability of care plans. Strengthening research and implementation of comprehensive, nurse-led approaches to CVD prevention may support the advancement of equitable, sustainable, and person-centered cardiovascular care.

## Data Availability

Not applicable. This review is based on previously published studies, all of which are cited in the References section. No new data were generated or analyzed during this study.

## Declarations

### AI-assisted technologies

The authors declare that no generative AI or AI-assisted technologies were used in the data collection or analysis. Only language editing support with AI-based tools was applied during manuscript preparation, under full author supervision and responsibility.

## Abbreviations

CVD: Cardiovascular disease
PRISMA: Preferred Reporting Items for Systematic Reviews and Meta-Analyses
SIGN: Scottish Intercollegiate Guidelines Network
